# Use of Passive Sensing in Psychotherapy Studies in Late Life: A Pilot Example, Opportunities and Challenges

**DOI:** 10.3389/fpsyt.2021.732773

**Published:** 2021-10-28

**Authors:** Jihui Lee, Nili Solomonov, Samprit Banerjee, George S. Alexopoulos, Jo Anne Sirey

**Affiliations:** ^1^Department of Population Health Sciences, Weill Cornell Medicine, New York, NY, United States; ^2^Weill Cornell Institute of Geriatric Psychiatry, Weill Cornell Medicine, White Plains, NY, United States

**Keywords:** depression, psychotherapy, mobile health, passive sensing, late life

## Abstract

Late-life depression is heterogenous and patients vary in disease course over time. Most psychotherapy studies measure activity levels and symptoms solely using self-report scales, administered periodically. These scales may not capture granular changes during treatment. We introduce the potential utility of passive sensing data collected with smartphone to assess fluctuations in daily functioning in real time during psychotherapy for late life depression in elder abuse victims. To our knowledge, this is the first investigation of passive sensing among depressed elder abuse victims. We present data from three victims who received a 9-week intervention as part of a pilot randomized controlled trial and showed a significant decrease in depressive symptoms (50% reduction). Using a smartphone, we tracked participants' daily number of smartphone unlocks, time spent at home, time spent in conversation, and step count over treatment. Independent assessment of depressive symptoms and behavioral activation were collected at intake, Weeks 6 and 9. Data revealed patient-level fluctuations in activity level over treatment, corresponding with self-reported behavioral activation. We demonstrate how passive sensing data could expand our understanding of heterogenous presentations of late-life depression among elder abuse. We illustrate how trajectories of change in activity levels as measured with passive sensing and subjective measures can be tracked concurrently over time. We outline challenges and potential solutions for application of passive sensing data collection in future studies with larger samples using novel advanced statistical modeling, such as artificial intelligence algorithms.

## Introduction

Major Depressive Disorder (MDD) in later life is a heterogenous condition characterized by high variability in biological and clinical features (1, 2). Individuals with MDD vary in their disease course with fluctuations in activity levels and mood during treatment (3). Most depression studies use rating scales administered once weekly to track change and these assessments do not capture granular time-sensitive changes (2, 4). Passive sensing data collection using smartphone sensors, such as pedometer, accelerometer, gyroscope, GPS, and microphone can capture fluctuations in daily functioning in real time (5, 6). The granularity and multimodal nature of passive sensing data can inform behaviors associated with outcomes and predict response more precisely (7).

While passive sensing has gained its popularity in mental health studies in youth and adult populations (8, 9), few studies examined its applicability on studying mental disorders in late life (10, 11). Even less is known about the use of passive sensing among older adults suffering from trauma and coping with chronic stress and high rates of depression and anxiety. This population is historically underserved and suffers from high rates of depression, anxiety, and post-traumatic stress. Insights from passive sensing data could help understand the heterogenous pattern of treatment response for each patient and thus guide personalization of these therapies to older adults' specific needs and circumstances.

Real-time routine tracking of movement and activity levels in depressed older adults—especially those suffering from trauma—can inform the study of engagement in behavioral activation (BA) psychotherapies that target increasing activity levels to reduce depression severity (12, 13). In these therapies patients are encouraged to engage in meaningful, rewarding activities, including increased time away from home, physical activity, and social interactions (13–15). We developed PROTECT, a behavioral activation and goal directed intervention for late life depression in elder abuse victims. PROTECT is intervention designed to reduce depression among elder abuse victims seeking elder mistreatment reduction services. It targets depressive symptoms by reducing victims' social isolation and increasing behavioral activation leading to a sense of agency and empowerment.

In this paper, we use case study examples from the PROTECT study (16) to present the potential utility of smartphone as a data collection tool in studies of psychotherapy for late-life depression. We examined the individual fluctuations in behavioral activation levels as well as trajectories of passive sensing measures during treatment course. We discuss opportunities and challenges and provide potential solutions and recommendations for future research.

## Methods

PROTECT psychotherapy includes 9 weekly sessions, where the therapist and the client work collaboratively toward realistic goals by implementing step-by-step action plans. PROTECT has shown to reduce depression severity and increase behavioral activation among elder abuse victims [See (17); See (16) for details]. Patients' reported levels of activity during the study was measured using the Behavioral Activation for Depression Scale (BADS) (18). During the 9-week treatment, BADS were measured at three time points; at baseline, weeks 6 and 9 (treatment end).

At recruitment, the participants consented to carry their smartphones during 9 weeks of intervention for passive sensing data collection and were informed of the types of data collected. They were given an iPhone if they did not own a smartphone. Participants received an instruction step-by-step booklet accompanied by technological training by research assistants on how to operate and use the smartphone. Therapists and research assistants provided ongoing technological support as needed. Participants were informed of the extent of passive sensing data collected from their smartphones and the data were securely stored and managed by using a server-based tracking program.

We focused on four passive sensing measures including step count, time spent at home, time in conversation and the number of times the phone was turned on (screen unlocks), and all measures were recorded daily. We utilized passive sensing data to infer an individual's daily living patterns. For example, higher daily step count reflects increased physical activity levels while more time spent at home may reflect greater isolation and lack of outside activity. More time in conversation may represent more social interaction with others. Finally, the number of screen unlocks is used as a utility measure, reflecting the level of engagement with the smartphone over time. The number of screen unlocks is also used to evaluate the granularity of passive sensing data. More screen unlocks is thought to indicate greater use of the phone and may increase data reliability and granularity (19).

One or more sensors were involved to define each passive sensing measure. For example, a pedometer was used to count the number of steps. Longitude and latitude coordinates were derived via Wi-Fi, cell phone towers and GPS. This location information was used to identify “home” and calculate time spent at home on a day. To protect participants' privacy, we did not record participants' actual geographic location but rather traveling patterns—moving east/west and north/south from an arbitrary reference location. Audio from a smartphone's microphone was sampled periodically to capture the participant's voiced signal. To protect user privacy, proprietary algorithms processed audio data in real time, destroying all contents and only capturing if and how long the participant was engaging in a conversation.

We preprocessed passive sensing data by removing unreliably low (or high) observations to prevent potential bias. Passive sensing recording is intrinsically dependent on the participant's level of engagement with their smartphone. Participants were asked to carry their phone at all times, but their level of engagement with their devices varied because participants might have not carried their phone during the day or their phones either was off/charging, was left at home, or had trouble authorizing the data collection. Heterogeneous levels of engagement across days within a single participant and across participants may result in different degrees of underestimation in passive sensing data and introduce biases if analyzed without addressing this issue. We implemented a 2-stage preprocessing algorithm for mobile health data. The first stage involved principal component analysis on the utility measures from the smartphone such as variability in the battery level and the number of raw observations of each passive measure within a day to quantify each participant's level of engagement. Days with extremely low level of engagement (the composite engagement score lower than the 30th quantile) were considered unreliable and labeled missing. The second stage used k-nearest neighbors algorithm to classify all unlabeled days. We did not impute the missing data; instead, we presented a smoothed curve to depict the overall trajectories of passive sensing data.

We explored the relation between individual reported activity on the behavioral activation measure (BADS) and passive data collected during the treatment. Overall, we examined the fluctuations in BADS scores and passive sensing measures on a within-person level. We visually inspected whether BADS scores and/or passive sensing measures increased or decreased compared to the individual's average levels and reported how the change in one coincided with the change in the other measures. Fluctuations of daily recorded passive sensing data were captured using a smooth local polynomial regression (LOESS) curve. This trajectory was visually compared with changes in BADS scores from baseline to weeks 6 and 9. As a result, we created individual-specific narratives to link observations from passive sensing data with their potential clinical implications.

## Pilot Examples

**Figure 1:**
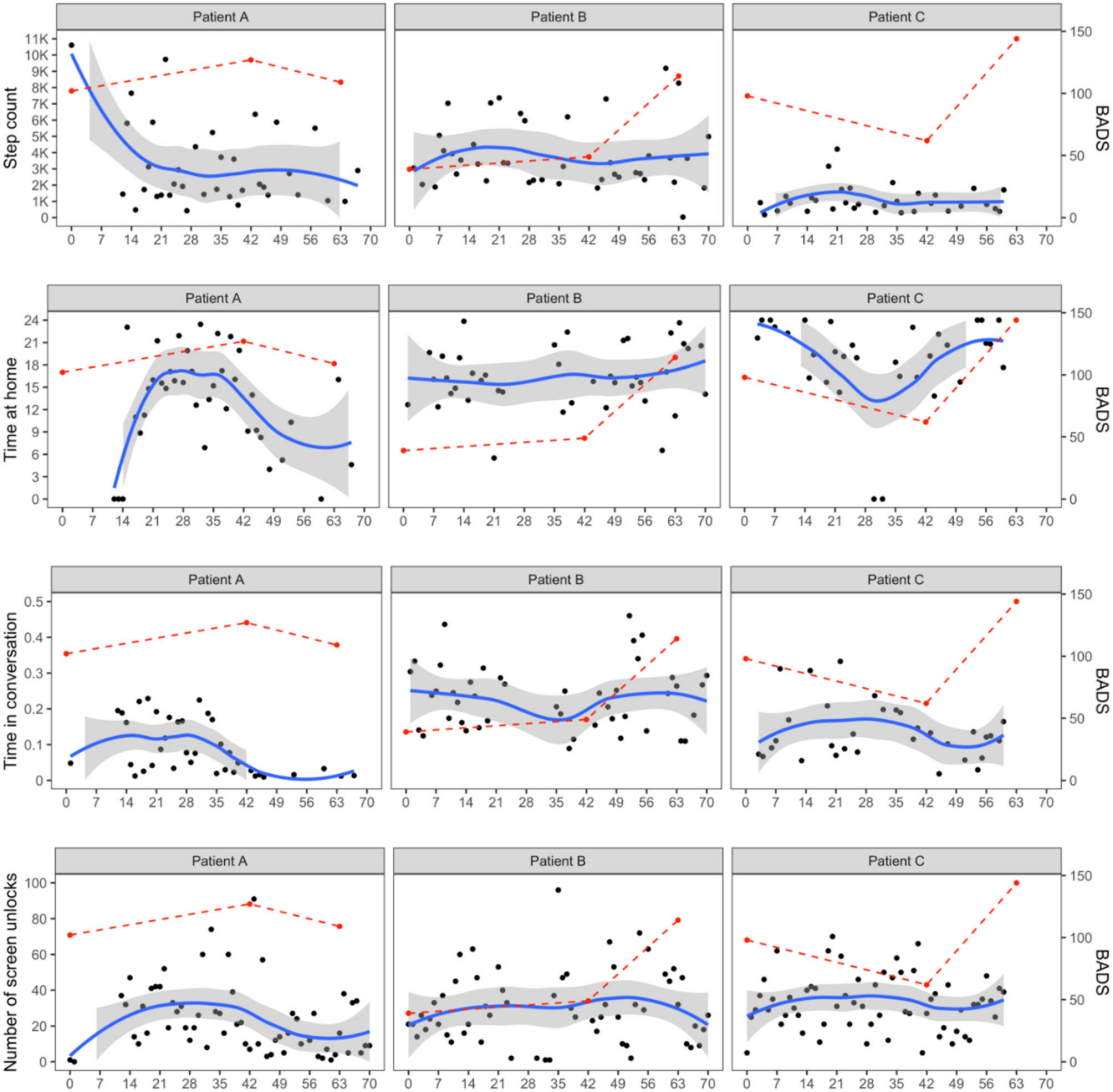
Passive sensing data fluctuations over treatment. BADS, Behavioral Activation for Depression Scale. Columns represent patients and rows represent different smartphone data (step count, time spent at home, time in conversation, and the number of screen unlocks). For all panels, the x-axis shows time in days. Points in black represent daily recorded smartphone data and a blue line with a shaded band is a smooth local polynomial regression (LOESS) curve with its 95% confidence interval. Points and a dashed line in red show the fluctuations in BADS scores from baseline, weeks 6 to 9 (end of treatment).

We selected three patients from a small pilot study comparing PROTECT with a referral control condition. These were representative pilot cases to illustrate the potential utility of passive sensing data among depressed older adults. The three patients were most compliant with study protocols of carrying around the smartphones during 9 weeks of treatment and thus produced the most granular passive sensing data. All three patients showed a clinical response, defined as 50% reduction in depressive symptoms on the Montgomery-Asberg Depression Rating Scale (MADRS) (20) score by the end of treatment (week 9). The study was approved by Weill Cornell Medicine's Institutional Review Board and all participants provided written consent for collection and processing of deidentified passive sensing data. Table 1 shows demographic and clinical characteristics of the subsample of three patients. Figure 1 shows the clinical and passive sensing data from these three patients.

**Table 1:** Demographic and clinical characteristics of the sample.

		**Patient A**	**Patient B**	**Patient C**
Treatment group		PROTECT	PROTECT	Referral
Age (years)		62	65	69
Gender		Male	Female	Female
Marital status		Separated	Divorced	Married
Living situation		Lives with Others	Lives Alone	Lives with Others
Ethnicity		Non-Hispanic	Hispanic	Non-Hispanic
Race		African American	White	African American
Religion		Other	Catholic	Catholic
Education (years)		14	12	14
Financial situation	Perception of financial status	Has just enough	Has just enough	Has just enough
	Annual Income	<9K	13K−16K	13K−16K
Abuse	Financial	Y		
	Verbal / Emotional	Y	Y	
	Physical	Y	Y	Y

Patient A is a 62-year-old Black man whose baseline MADRS score was 27. He experienced financial, verbal/emotional, and physical abuse. He showed significant improvement in depression during treatment with a MADRS score of 6 at treatment end. We were not able to obtain passive sensing data from his smartphone in the first 2 weeks of the study, likely due to technical difficulties or need of reminders to carry the phone at all times. However, following this initial period, his consistently high screen unlocks suggested high engagement with his smartphone throughout the intervention after the first 2 weeks. Patient A began and ended therapy with relatively low levels of behavioral activation (Intake BADS = 102; Week 9 BADS = 109). These low activity levels correspond with less time spent in conversation at the beginning and end of treatment and decrease in step count by Week 9. At Week 6, Patient A reported the highest activity levels (BADS = 127). This increase corresponded with more time spent at home and more time in conversation, which may indicate this patient engaged in helpful activities and conversations with others at home during mid-treatment.

Patient B is a 65-year-old White Hispanic woman who was divorced and lived alone with a history of verbal/emotional and physical abuse. She reported severe major depression (MADRS score of 33 at baseline) and extremely low levels of behavioral activation (BADS = 39) at the start of treatment. Patient B was consistently engaged with her phone, as reflected by high screen unlocks throughout treatment. By Week 6, the patient reported an increase in behavioral activation levels (BADS = 49). Patient B showed consistently high step count (averaging 4,000 steps a day), spent time in conversation and was away from home throughout treatment. This high engagement in multiple activities may have contributed to her significant reduction in depression (MADRS at Week 9 = 15), as well as meaningful increase in levels of behavioral activation (BADS = 114).

Patient C is a 69-year-old Black woman who reported physical abuse with moderate depression (MADRS = 23) and relatively high levels of behavioral activation at intake (BADS = 98). Patient C maintained high levels of screen unlocks reflecting consistent engagement with her phone. However, low step count and time spent in conversation throughout treatment reflected low outside and social activity levels. Nevertheless, her BADS score increased to 114 by treatment end. For this patient, more time at home corresponded with higher levels of behavioral activation at the beginning and end of treatment. During Week 6, the patient did not spend much time at home, suggesting potential disruption to her usual routine. It may have contributed to the lower behavioral activation (BADS = 62). This also suggests she may have engaged in pleasurable activities at home.

## Opportunities and Challenges

Passive sensing data offer the potential to observe daily activity between standardized assessments of behavioral activation and changes in depression severity (21). Our data illustrate the individual level variation observed among three patients who showed improved depression during PROTECT treatment. All three patients showed clinically significant response and were engaged with the smartphone during the study. However, the figures illustrate variability in passive sensing data and behavioral activity level reports within-patient over time as well as between-patient differences. Real-time assessment of individuals in their natural environments maximizes ecological validity and the granularity of smartphone data can capture detailed fluctuations of behavior over the study period (4). The variability observed may also reflect the different ways that patients become activated as part of the therapy.

Multimodal data can provide a more nuanced understanding of behavioral patterns for each individual. The passive sensing data provide an opportunity for digital phenotyping, i.e., moment-by-moment quantification of the individual-level human phenotype *in-situ* (22, 23). Passive sensing can reflect changes in physical activity and time spent outside (24), which may correlate with mental health outcomes, such as loneliness and social isolation (25), as well as anxiety, stress and depression (26). In our project, step count and time spent at home were used to quantify participants' daily activity. By considering these measures simultaneously we discerned days with high activity level at or around home from those days with a greater travel diameter. Additional information regarding planned goals and the types of activities patients engaged in while at home or outside could expand our understanding of clinical meaning of passive sensing data on an individual level. Further, future work could investigate associations between activity levels and loneliness and social isolation, which is prevalent among elder abuse victims. Collection of these data in a large sample may contribute to the understanding of behavioral patterns associated with treatment response and guide development of personalized treatments (27, 28).

Unique characteristics of passive sensing introduce a new area of data analyses methods. Although we did not apply advanced analytic methods in this small, classic statistical methods such as mixed effects models and generalized estimation equation as well as pre vs. post hypothesis tests can be used to analyze the temporal changes in passive sensing data (29–32). Creating a platform that streamlines the passive sensing data collection, management and analysis allows to collect a bigger sample (23), and passive sensing data in a large sample provides ample opportunities to develop and implement sophisticated statistical methods and machine learning algorithms (32) for suicide prediction, for example. Types of machine learning and artificial intelligence algorithms for passive sensing data range from feature extraction and selection (33), gradient boosting (6), to artificial neural networks (34). Large sample data might help ferret out what activities are most frequently associated with increase in activity levels, behavioral activation reports and improvement in depression.

A challenge of implementation of passive data is reliance on the engagement with the smartphone. The patients we presented consistently used their smartphone. However, many elder abuse victims may struggle to maintain high levels of engagement. These individuals often struggle with chronic and acute trauma and are more likely to be members of marginalized minorities, come from lower socioeconomic background, and experience medical burden and disability (35). Previous studies have documented a digital divide within the older adult population, with those from lower socioeconomic status and less resources least likely to adapt to technology (36–38). However, studies have shown that technology use among older adults has increased dramatically over the past two decades (37). In our case study examples, we observed from Patient A that it may take a while for older adults to get used to using the mobile devices, but they could adapt to use the new technology and provide useful passive sensing data (39). Nonetheless, tailoring technology to the older adults' specific needs and circumstances can significantly enhance passive sensing data quality, validity and accuracy (40–42). To protect participants' privacy, we did not collect the content of conversations or the specific locations visited when participants left their home. Data on content could elucidate the affective valence of conversations and their potential effect on outcome. Social interactions with supportive others are especially therapeutic (15). However, it is also possible that elder abuse victims spent time speaking with supportive others, or alternatively with the identified abuser. Similarly, we do not know whether participants who left their home engaged in pleasurable activities aligned with their treatment goals, or activities that may have increased distress. Further research is needed to examine the qualitative nature of passive sensing data collected to increase clinical interpretability.

In summary, passive data tracking can provide nuanced granular data on activity and engagement patterns over time. Despite substantially growing interest in incorporating mobile technology to mental health studies in recent years, the extent of technology used for continuous monitoring of older adults has been relatively limited to environmental such as in-home sensors (11). To our knowledge, this is the first study which used passive sensing data from a population of elder abuse victims. If integrated with clinical response trajectories, passive sensing data can improve identification of personalized interventions leading to increased activity and well-being among older adults (10). However, the reliability of smartphone data is dependent on the participant's active and sustained engagement with smartphones (43). Challenges include low perceived ease of smartphone use and the lack of technological support tailored to older adults' needs. Potential solutions include implementing changes in mobile technology based on older adults' needs and preferences and use of wearable devices. Future work will investigate relationships between activity levels measured by passive sensing and treatment outcomes in larger samples using advanced statistical approaches.

## Data Availability Statement

The datasets presented in this article are not readily available because data could be made available by the authors in compliance with the funder's data sharing policy. Requests to access the datasets should be directed to jsirey@med.cornell.edu.

## Ethics Statement

The study was approved by Weill Cornell Medicine's Institutional Review Board. The patients/participants provided their written informed consent to participate in this study. Written informed consent was obtained from the individual(s) for the publication of any potentially identifiable images or data included in this article.

## Author Contributions

JL, NS, and SB contributed to the analyses. GA, JS, and SB contributed to study design. All authors contributed to the article and approved the submitted version.

## Funding

This study was supported by P50 MH113838 (GA) and K23 MH123864 (NS).

## Conflict of Interest

The authors declare that the research was conducted in the absence of any commercial or financial relationships that could be construed as a potential conflict of interest.

## Publisher's Note

All claims expressed in this article are solely those of the authors and do not necessarily represent those of their affiliated organizations, or those of the publisher, the editors and the reviewers. Any product that may be evaluated in this article, or claim that may be made by its manufacturer, is not guaranteed or endorsed by the publisher.
